# Comparative Study of The Yield and Physicochemical Properties of Collagen from Sea Cucumber (*Holothuria scabra*), Obtained through Dialysis and the Ultrafiltration Membrane

**DOI:** 10.3390/molecules26092564

**Published:** 2021-04-28

**Authors:** Suryani Saallah, Jumardi Roslan, Flavian Sheryl Julius, Sharinee Saallah, Umi Hartina Mohamad Razali, Wolyna Pindi, Mohd Rosni Sulaiman, Khairul Faizal Pa’ee, Siti Mazlina Mustapa Kamal

**Affiliations:** 1 Biotechnology Research Institute, Universiti Malaysia Sabah, Jalan UMS, Kota Kinabalu 88400, Sabah, Malaysia; suryani@ums.edu.my; 2 Faculty of Food Science and Nutrition, Universiti Malaysia Sabah, Jalan UMS, Kota Kinabalu 88400, Sabah, Malaysia; flaviansheryl@gmail.com (F.S.J.); sharineesaallah@gmail.com (S.S.); umi.hartina@ums.edu.my (U.H.M.R.); woly@ums.edu.my (W.P.); rossulma@ums.edu.my (M.R.S.); 3 Section of Food Engineering Technology, Malaysian Institute of Chemical and Bioengineering Technology, Universiti Kuala Lumpur, Alor Gajah 78000, Malacca, Malaysia; khairulfaizal@unikl.edu.my; 4 Department of Process and Food Engineering, Faculty of Engineering, Universiti Putra Malaysia, UPM Serdang, Shah Alam 43400, Selangor, Malaysia; smazlina@upm.edu.my

**Keywords:** sea cucumber, collagen, dialysis, ultrafiltration membrane, physicochemical properties

## Abstract

Collagen was extracted from the body wall of sea cucumber (*Holothuria scabra*) using the pepsin-solubilized collagen method followed by isolation using dialysis and the ultrafiltration membrane. The yield and physicochemical properties of the collagen obtained from both isolation methods, denoted as D-PSC and UF-PSC, were compared. The ultrafiltration method affords a higher yield of collagen (11.39%) than that of the dialysis (5.15%). The isolated collagens have almost the same amino acid composition, while their functional groups, referred to as amide A, B, I, II, and III bands, were in accordance with commercial collagen, as verified by Fourier Transform Infrared (FT-IR) spectroscopy. The UV-Vis absorption peaks at 240 nm and 220 nm, respectively, indicated that the collagens produced are type-I collagen. The D-PSC showed interconnecting sheet-like fibrils, while the UF-PSC exhibited a flaky structure with flat-sheets arranged very close to each other. The higher yield and comparable physicochemical properties of the collagen obtained by ultrafiltration as compared with dialysis indicate that the membrane process has high potential to be used in large-scale collagen production for food and pharmaceutical applications.

## 1. Introduction

Sea cucumbers are marine animals that belong to the phylum Echinodermata from the class *Holothuridea*. As the name suggests, they have soft, elongated bodies with leathery skin, resembling a cucumber. About 1250 species of sea cucumber have been identified, particularly in the benthic and deep seas across the world. Among them, *Holothuria* is the most important sea cucumber species with high economic and trade values. Most of *Holothuria* species can be found in South-East Asian countries, including Malaysia, with the most harvested species being *Holothuris scabra* (sandfish), *Holothuria nobilis* (black teatfish), and *Holothuris fuscogilva* (white teatfish) [1,2].

Sea cucumbers have long been used by Asian people as a dietary delicacy and a traditional remedy for healing various internal and external wounds. The medicinal and therapeutic functions of sea cucumbers are mainly ascribed to the presence of biologically active substances. Various bioactive compounds isolated from several sea cucumber species have shown useful pharmacological functions such as collagen, acid mucopolysaccharide, and triterpene glycoside [3]. The body wall of sea cucumbers consists mainly of collagen, an essential protein component in connective tissues [4]. It is distinct from other proteins where the molecule comprises three polypeptide chains, which form a unique triple helical structure. Collagen has a wide range of applications in the biomedical, pharmaceutical, cosmetic, and food industries [5]. 

Extraction of collagen from different species of sea cucumber has been reported in a number of studies, including *Stichopus japonicus* [4,6,7], *Holothuria parva* [8], *Stichopus vastus* [9], *Bohadschia bivitatta* [10], and *Parastichopus californicus* [11]. Most of the studies have employed the pepsin solubilized collagen method to extract the collagen, followed by isolation through salt precipitation and dialysis. However, this conventional isolation method is laborious, time-consuming, prone to protein denaturation, and difficult to scale-up for industrialization. Therefore, searching for an effective method to enhance collagen recovery is crucial.

Membrane technology is seen as the appropriate technique for this purpose, especially the ultrafiltration (UF) membrane, and it has been successfully used for concentration and purification of collagen from various sources [12,13,14,15,16]. UF could offer a low-cost, high-throughput, and scalable collagen isolation method. Most importantly, the isolation time can be shortened to ~3 h in comparison with the conventional method using dialysis, which usually takes 1–3 days to complete. In some cases, repeated salt precipitation and dialysis are required, thereby increasing the time required even more (up to 6 days) [16].

Although the application of the UF membrane for collagen isolation has been reported in many studies, these studies mainly emphasized the development of membrane material simply used for collagen separation and purification. As for collagen extracted from sea cucumber (*Holothuria scabra*), to our knowledge, a comparison between the yield and characteristics of collagen isolated through dialysis and the UF process has never been reported. Therefore, the present study aims to investigate the effect of different isolation methods on the yield and physicochemical properties of *Holothuria scabra* collagen. Information regarding the yield, amino acid composition, purity and physicochemical properties of collagen obtained from both methods is of fundamental importance to ensure a high yield of collagen can be obtained without compromising its structural integrity and intrinsic properties, as well as to determine potential applications of the collagen.

## 2. Results and Discussion

### 2.1. Yield, pH and Color of Collagen

In the present study, collagen from sea cucumbers (*Holothuria scabra*) was extracted using the Pepsin-Solubilized Collagen (PSC) method and isolated using two different approaches, namely dialysis and the ultrafiltration (UF) process. The collagen obtained were denoted as D-PSC and UF-PSC, respectively. Table 1 shows the yield (calculated based on a dry weight basis), pH, and the PSCs’ color. The collagen yield for UF-PSC (11.39 %) was significantly (*p* < 0.05) higher than for D-PSC (5.15 %), which indicated that separation of collagen using the UF membrane is more efficient compared with the conventional method (dialysis). 

In basic terms, UF is a pressure-driven system that provides means of removing undesired components, such as protein with lower molecular weight and inorganic salt, from the collagen solution. As for dialysis, separation relies on a concentration gradient. In this experiment, UF requires 12 h to complete the separation, while the conventional dialysis method requires a slower process (1–3 days). Therefore, UF was proven as an effective and rapid approach for collagen separation with a high yield. It corresponds to findings reported by Chen et al. [16], where the UF process offers several advantages over the dialysis method for collagen isolation in terms of time, processing volume and quality of collagen. The yield of PSCs obtained in this study is comparable to *Holothuria parva* (7%) [8] but lower than the yield of PSCs from other types of sea cucumber, such as *Stichopus vastus* (21.3%) [9], *Stichopus japonicus* (26.6%) [17], and *Parastichopus californicus* (20%) [11]. The variation of PSC yield of different species of sea cucumber is mainly attributed to the degree of crosslinking of peptides at the telopeptide region and the difference in the sea cucumber body wall composition [8,10].

The pH value plays a vital role in collagen formulation, especially for commercialization [18]. According to Alhana et al. [19], the best collagen quality should be in the pH range of 6.5 to 8 [19]. As shown in Table 1, the pH values for D-PSC and UF-PSC were 3.65 ± 0.42 and 6.23 ± 0.49, respectively. Based on this result, it was found that collagen isolation using the UF membrane managed to produce a neutral collagen characteristic, which indicates that the UF membrane is more effective in removing salt from the solution than dialysis. The effectiveness of the UF membrane most probably due to the pressure-drive used by the system, which provides more force to remove undesired components such as acetic acid and salt through the membrane in a short time and eventually produces a neutral collagen solution.

Color measurement is essential in determining collagen quality attributes, particularly in the food industries, and it influences consumer’s choices and preferences. The color of collagen was evaluated based on ‘L’ (lightness) value, ‘a’ (redness), and ‘b’ (yellowness). The high ‘L’ values obtained for both PSCs indicate a whiter attribute of the samples, thus eliminating the need for decolorization for commercial application. The D-PSC was slightly lighter than the UF-PSC, while the redness and yellowness were not significantly different.

### 2.2. Amino Acid Composition of Collagen

D-PSC and UF-PSC were further analyzed for their amino acid composition, and the results are shown in Table 2. Both the D-PSC and UF-PSC were rich in glycine, glutamic acid, proline and alanine. These findings are in accordance with the amino acid composition reported by several researchers for different species of sea cucumber [4,6,8,11]. Interestingly, UF-PSC has shown relatively higher content for all the main amino acids (glycine, glutamic acid, proline and alanine) than D-PSC. High contents of glycine, proline and alanine suggested the presence of collagen [7]. It was reported that glycine was present in every third residue in the collagen, forming a tri-peptide unit. The tri-peptide sequence was mainly occupied with proline and hydroxyproline (imino acids), which play an important role in stabilizing the collagen helices [20].

The imino acid (proline + hydroxyproline) contents of D-PSC is significantly higher (*p* < 0.05) than UF-PSC, with values of 18.47% and 17.41%, respectively. The slightly lower imino acid content for UF-PSC might be due to the tendency of collagen, particularly its amino acids, to interact with the membrane surface. Accumulation of collagen on the membrane surface could lead to membrane fouling and consequently influence imino acid recovery. This result agrees with the finding reported by Shen et al. [12], where many proteins are very surface active and can be adsorbed onto any polymeric membrane and interact with many other components in the feed. The imino acid contents of D-PSC and UF-PSC obtained here were higher than those reported for Stichopus japonicus [6,7] and Parastichopus californicus [11] but slightly lower than Stichopus japonicus, as reported by Zhu et al. [4]. An increased imino acid content throughout the collagen matrix formed more stable collagen. Additionally, the hydroxyproline ratios to proline for D-PSC and UF-PSC were 0.77 and 0.63, respectively, which indicate that the structure of PSC from Holothuria scabra is closer to type I collagen [8]. The imino acid content in the collagen of a species may vary depending on its living habitat [11] and the collagen extraction condition [20]. Collagen with high imino acid content is desirable for industrial applications due to its high thermal stability. The lowest amino acid composition found for both UF-PSC and D-PSC was cysteine, with values of 0.18% and 0.13%, respectively.

### 2.3. Characterization of Collagen

**Figure 1:**
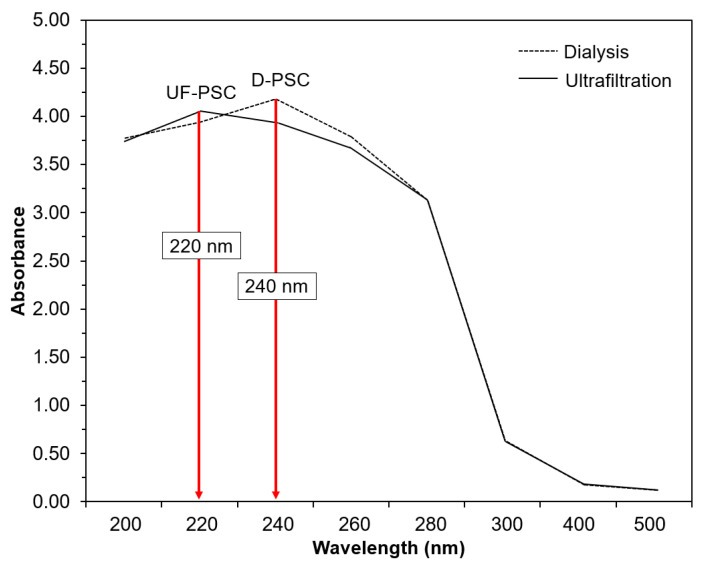
UV-Vis spectra of collagen from sea cucumber (*Holothuria scabra*) isolated through dialysis and the ultrafiltration membrane.

UV-Visible spectrophotometric analysis was performed to determine collagen’s purity based on the absorption at a specific wavelength [6]. The UV-Vis spectra of D-PSC and UF-PSC are shown in Figure 1. It was found that UF-PSC and D-PSC exhibit a maximum absorbance at 220 nm and 240 nm, respectively. According to Cui et al. [6], the collagen fibrils exhibited maximum absorbance at 220 nm was identical to type 1 collagen. A slightly higher absorbance value was obtained for D-PSC, confirming the high purity of UF-PSC. In addition, the purity of collagen obtained is further supported by the result for which there is no other absorption observed at around 280 nm, which is believed to contain aromatic amino acids (Tyrosine and Phenylalanine) [6,8].

**Figure 2:**
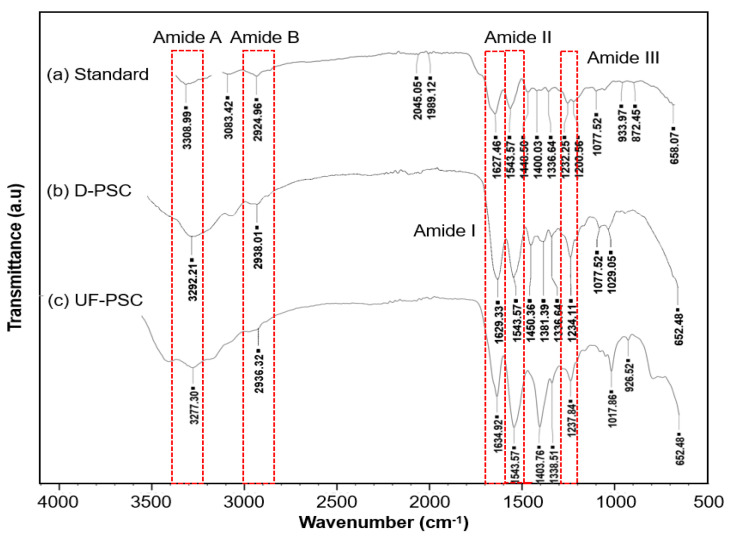
FTIR spectra of collagen (**a**) standard collagen (calf skin); (**b**) collagen isolated using dialysis, D-PSC; and (**c**) collagen isolated using ultrafiltration, UF-PSC.

The FT-IR spectra of D-PSC and UF-PSC are shown in Figure 2. Collagen from calf skin was used as a standard to identify the characteristic peaks of pure collagen. Five major bands, which are characteristic of amides, including amides A, B, I, II, and III, were found for calf skin collagen type I at wavelengths of 3308, 2924, 1627, 1,543, and 1232 cm^−1^, respectively. All these characteristic peaks are present in the D-PSC and UF-PSC spectra but with slightly shifted values. The absorption bands observed for both the PSCs were almost similar to the absorption bands reported for collagens isolated from other sea cucumber species, such as *Stichopus vastus* [9], *Stichopus japonicas* [4], and *Holothuria parva* [8]. The amide A is associated with the N-H stretching frequency, which usually occurs in the range of 3400–3440 cm^−1^ [21]. However, when the NH group of a peptide is involved in hydrogen bonding, the position is shifted to lower frequencies [3,9]. There was a lower frequency in the amide A spectrum for both D-PSC and UF-PSC, indicating the involvement of NH groups of collagens in hydrogen bonding. Amide B bands of D-PSC and UF-PSC were found at 2938 cm^−1^ and 2936 cm^−1^, respectively, which is related to the asymmetrical stretching of CH_2_ [8]. The bands appearing at 1629 cm^−1^ for D-PSC and 1634 cm^− 1^ for the UF-PSC were assigned to amide I band, which was mainly associated with the stretching vibration of the carbonyl groups along the peptide backbone. Amide I band is the most important factor in understanding the secondary structure of protein molecules. The amide II band around 1543 cm^-1^ is associated with N-H deformation. The existence of collagen triple helical structures was confirmed by the presence of amide III bands at around 1234 cm^−1^ and 1237 cm^−1^ for both D-PSC and UF-PSC, respectively [4,8,16]. Other minor bands that have a presence in both D-PSC and UF-PSC were also characterized by the carboxyl group, which can be found at around 1403–1450 cm^−1^ [12,22]; CH_2_ deformation at 1338 cm^−1^ [16]; and the ester bond, which appears at 1077 cm^−1^ [22].

**Figure 3:**
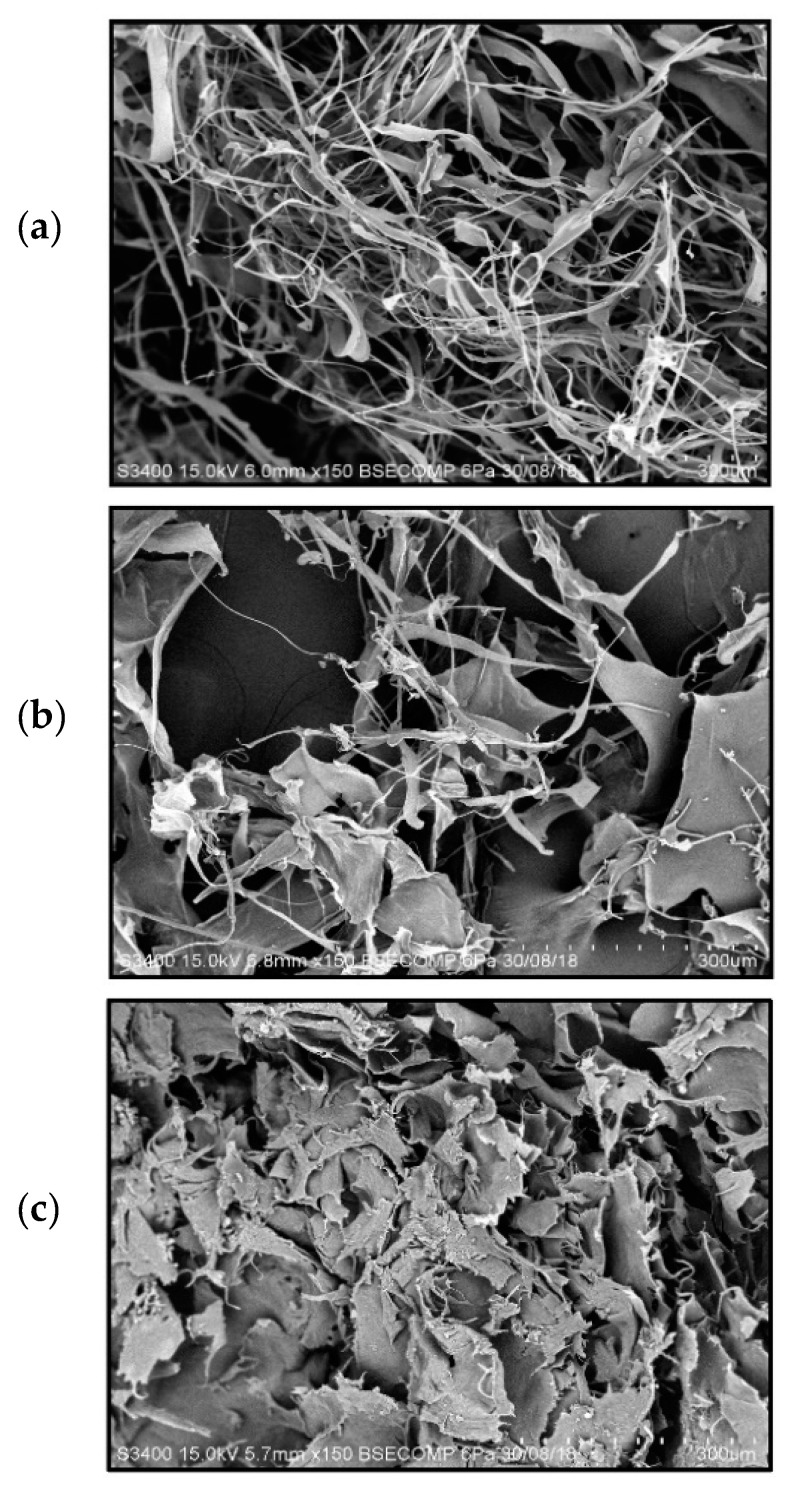
SEM images of collagen (**a**) standard Collagen (calf skin), (**b**) D-PSC, and (**c**) UF-PSC.

Figure 3 shows the scanning electron microscope (SEM) images of collagens from calf skin collagen (a), D-PSC (b), and UF-PSC (c). It was observed that the collagen from calf skin demonstrated bundles of circumference fibrils networks, irregular and dense pleated structures, which were characterized as type I collagen. According to Zhang et al. [23], lyophilized collagen from calf skin would have a loose and porous structure. SEM analysis for D-PSC has shown a less fibrils network with flat sheets and some flat interconnecting sheet-like fibrils. This observation was in accordance with the study reported by Siddiqui et al. [10]. However, UF-PSC has shown a different morphology, where collagen exists as flat sheets arranged very close to each other. Collagen fibers occur naturally in a fibrillar or uniform porous network and are sometimes arranged in parallel to give great strength, or they may be highly branched and disordered as in skin [23]. The difference in the morphological structure of collagen obtained from dialysis and UF methods could be explained by the mechanism through which the isolation process occurs, which affects the arrangement of collagen molecules during freeze drying. Dialysis involves diffusion of solute based on the concentration gradient until reaches an equilibrium. As for UF, the separation is driven by hydrostatic pressure, which could retain the collagen from passing through the membrane and facilitate the separation of unwanted protein of lower molecular weights and inorganic salts from the collagen solution. As the process continues, the collagen accumulated on the membrane surface, and highly concentrated collagen (retentate) was obtained when the separation was completed. When subjected to freeze-drying, the collagen molecules might undergo rearrangement due to ice-crystal formation, thus producing a flat-sheet structure, as reported by Sun et al. [24].

## 3. Materials and Methods

### 3.1. Sample Preparation

Sea cucumber (*Holothuria scabra*) was obtained live from a local sea cucumber farm in Kota Belud, Sabah, Malaysia. Upon arrival at the laboratory, the sea cucumber was immediately processed by removing the viscera. Then, the sea cucumber’s body wall was cut into a small piece (1 cm × 1 cm) prior to blending using a high-speed grinder. The minced sea cucumber was packed in small polyethylene plastic bags, frozen, and stored at −20 until further use.

### 3.2. Extraction of Collagen

Collagen from the body wall of sea cucumber (*Holothuria scabra*) was extracted according to the method described by Abedin et al. [9] with slight modifications. Briefly, 100 g of minced sea cucumber body wall was subjected to two times washing with deionized distilled water, followed by continuous stirring for 30 min. After that, the suspension was decanted, and 1 L of ethylenediaminetetraacetic acid (EDTA) solution (4 mM) containing Tris-HCl (0.1 M, pH 8.0) was added and stirred continuously for 24 h. These processes were repeated but with the use of 1 L of deionized water to replace the EDTA/Tris-HCl solution. After removing the water-insoluble components from the mixture using a cheesecloth, the free collagen fibrils were centrifuged at 12,000× *g* for 30 min. The precipitate obtained after centrifugation was treated with 20 volumes of NaOH (0.1 M), followed by 72 h of stirring to dissolve the non-collagenous components. The collagen was then recovered by 30 min centrifugation at 12,000× *g*. The insoluble residue was washed several times with deionized distilled water until a neutral pH was obtained, before it was subjected to solubilization with 20 volumes of 0.5 M acetic acid containing 5 gL^−1^ of pepsin. The process was left to run for 48 h with continuous stirring. The precipitate was recovered by centrifugation (25,000× *g*, 60 min) and treated with mixture of acetic acid/pepsin mixture to extract the remaining collagen, as described above. The Pepsin Solubilized Collagen (PSC) obtained was then isolated using conventional dialysis and ultrafiltration methods. Extraction and isolation were conducted in triplicates, and the results were reported in average values.

### 3.3. Isolation of Collagen Using Conventional Method

Conventional collagen isolation was performed by salt-precipitation and the dialysis method. Briefly, the PSC was subjected to a salting-out process with 0.8 M of NaCl. The solution was stirred for 24 h prior to 30 min of centrifugation at 12,000× *g* to collect the crude collagen. The crude collagen was then dissolved in 0.5 M acetic acid and dialyzed using a dialysis tube (Cellulose Membrane, D9777) with a molecular weight cut-off (MWCO) of 14 kDa, against 0.1 M acetic acid, followed by deionized water for 3 days. The purified PSC (D-PSC) was freeze-dried and stored in a refrigerator until further use.

### 3.4. Isolation of Collagen Using the Ultrafiltration Membrane

Isolation of collagen using the UF membrane was conducted using a dead-end UF membrane system (Amicon model 8200 stirred ultrafiltration cell, Amicon Corp., Danvers, MA). A flat sheet of regenerated cellulose (RC) with a molecular weight cut-off (MWCO) of 10 kDa (Millipore, PLGC 06210) was used as the membrane. The stirred UF cell is equipped with a 4.9 cm bar impeller that was magnetically driven by a stirring hot plate, positioned about 1.5 mm above the membrane, as shown schematically in Supplementary Figure S1. A phototachometer was used to monitor the stirring speed. Before the isolation process, the precipitated collagen was dissolved in 200 mL of 0.5 M acetic acid and transferred to the ultrafiltration cell. The separation was conducted at a pressure of 2.0 bar and stirring speed of 500 rpm for 12 h. The concentrated collagen (UF-PSC) was then collected and freeze-dried before further use.

### 3.5. Yield of Collagen

The yield of collagen obtained from both the dialysis (D-PSC) and UF (UF-PSC) methods was calculated based on the following equation [25]:

(1) $$\mathrm{Yield}\;\left(\%\right)=\frac{\mathrm{Weight}\;\mathrm{of}\;\mathrm{dry}\;\mathrm{lyophilised}\;\mathrm{collagen}\;\left(g\right)}{\mathrm{Weight}\;\mathrm{of}\;\mathrm{initial}\;\mathrm{dry}\;\mathrm{of}\;\mathrm{body}\;\mathrm{wall}\;\left(g\right)}\times 100$$

### 3.6. pH Determination

The pH of the hydrolyzed collagen was measured using a pH meter (Model340, Mettler-Toledo, Zürich, Switzerland). Briefly, 1.0% of the collagen solution was dissolved in distilled water at an ambient temperature, and the pH value was taken using a pH probe. All measurements were performed in triplicate.

### 3.7. Color Determination

Color analysis was performed using a HunterLab Colorimeter (HunterLab, Model CX 2379). L*, a*, and b* values, which represent lightness, chromatic scale from green to red, and chromatic scale from blue to yellow, respectively, were measured. Experiments were conducted in triplicates, and the results were reported as an average.

### 3.8. UV-Visible Measurement

The UV absorption spectra of the isolated collagen samples were recorded individually by a spectrophotometer, following the method described by Zhu et al. [4] with slight modifications. The collagen samples were prepared by dissolving 10 mg of collagen in 10 mL of 0.5 M acetic acid. The resulting solutions were placed into a quartz cell, and the absorption wavelengths were measured in the range of 200 to 500 nm.

### 3.9. Fourier Transform Infra-Red (FTIR) Spectroscopy

FTIR spectrophotometer (Thermo Scientific, USA) was used to record the FTIR spectrum of the isolated PSCs to obtain further information on the collagen secondary structure with regards to different isolation methods. The spectrum was recorded from 500 to 4000 cm^−1^, with a resolution of 4 cm^−1^ and average scans of 64 [9].

### 3.10. Determination of Amino Acid Compositions

The amino acid compositions of the isolated PSC were identified using HPLC, following the method described by Jamilah et al. [20]. The isolated PSCs were first hydrolyzed with 5 mL of HCl (6 N) at 110 °C for 24 h. Then, 4 mL of internal standard α-aminobutyric acid (AABA) was added to the residue, and volume of the mixture was made up to 100 mL with deionized water. Derivatization was carried out using an AccQ-Fluor Reagent kit (Waters Co., Milford, MA, USA). Then, 10 µL of standard solution or the hydrolyzed samples was mixed with 70 µL of borate buffer and 20 µL of AccQ reagent and transferred to autosampler vials. The mixture was then incubated for 10 min at 55 °C. Peak separation was carried out by injecting 5 µL of the mixture into an AccQ Tag RP-column (3.9 × 150 mm, Waters Co., Milford, USA) using a gradient run. The eluent system consisted of two components: 40% AccQ Tag concentrate as Eluent A and 60% acetonitrile as Eluent B. The gradient condition was programmed as follows: 100% A in 0.5 min, 98% A in 14.5 min, 90% A in 4 min, 87% A in 13 min, 65% A in 2 min, and 0% A in 3 min, followed by 100% A for 13 min at a flow rate of 1 mL/min. Detection was achieved using a fluorescence detector (FD) (Waters 2475, Waters Co., Milford, USA). The amino acids’ determination was carried out using a Waters Auto analyzer (Waters 2690/5, Waters Co., Milford, USA).

### 3.11. Scanning Electron Microscopy (SEM)

Scanning Electron Microscope (SEM) was used to observe the collagen microstructure. Freeze-dried PSCs were cut into small pieces and mounted on a stub using double-coated conductive carbon tape. Before the analysis, the samples were sputter-coated with a thin layer of gold in a vacuum evaporator (Bal-Tech, SCD005, Balzers, Germany). The analysis was performed at an accelerating voltage of 15 kV.

### 3.12. Statistical Analysis

Data were analyzed using Statistic SPSS software version 2.0 for ANOVA and a *t*-test comparison test.

## 4. Conclusions

Pepsin-solubilized collagen from the body wall of sea cucumber (*Holothuria scabra*) has been successfully isolated using dialysis and an ultrafiltration membrane. UF-PSC exhibits a higher collagen yield (11.39%) than D-PSC (5.15%), indicating the efficiency of the process to recover collagen. Both collagens, isolated using dialysis and UF membrane, were further characterized in terms of amino acid composition, UV-Vis spectrum, FT-IR and morphology. A slightly higher amino acid concentration was found in UF-PSC compared with D-PSC. Based on UV-VIS spectrum analysis, it was found that both UF-PSC and D-PSC exhibit maximum absorbance at 220 nm and 240 nm, which is similar to type 1 collagen. The FT-IR spectra have shown that both collagens consist of five major bands characterized as amides, including amides A, B, I, II, and III. SEM analysis has shown a less fibrils network with flat sheets and some flat interconnecting sheet-like fibrils for D-PSC, and a flat sheet arrangement was observed for UF-PSC. Based on the overall findings, the ultrafiltration process had advantages over the dialysis method for collagen isolation in terms of time efficiency, higher yield and comparable quality. The results indicated that relatively high purity of collagens was obtained from the body wall of sea cucumbers (*Holothuria scabra*). They could be an alternative source of mammalian collagen and have potential applications in various fields, including food, pharmaceutical, nutraceutical and cosmetics.

## Figures and Tables

**Table 1:** Yield, pH and color of D-PSC and UF-PSC.

Properties	D-PSC	UF-PSC
Collagen Yield (%)	5.15 ± 0.30 ^b^	11.39 ± 2.86 ^a^
pH	3.65 ± 0.42 ^b^	6.23 ± 0.49 ^a^
Color		
L* (lightness)	80.90 ± 1.94 ^a^	77.44 ± 1.67 ^b^
a* (green to red)	0.37 ± 0.23 ^a^	0.48 ± 0.45 ^a^
b* (blue to yellow)	3.81 ± 1.50 ^b^	4.24 ± 1.58 ^b^

All data expressed as mean ± S.D. (n = 3). Similar superscript letters within each row were not significantly different (*p* > 0.05).

**Table 2:** Amino acid composition of D-PSC and UF-PSC.

Amino Acid Composition	D-PSC	UF-PSC
Glycine	17.86 ± 0.06	18.39 ± 0.10
Glutamic acid	14.67 ± 0.10	15.77 ± 0.04
Alanine	10.15 ± 0.05	10.85 ± 0.08
Proline	10.42 ± 0.12	10.68 ± 0.03
Arginine	9.79 ± 0.02	9.05 ± 0.01
Aspartic acid	8.43 ± 0.10	9.04 ± 0.07
Hydroxyproline	8.05 ± 0.06	6.73 ± 0.10
Threonine	4.37 ± 0.03	4.19 ± 0.01
Serine	3.73 ± 0.05	3.52 ± 0.08
Leucine	3.07 ± 0.03	2.98 ± 0.01
Valine	2.86 ± 0.10	2.80 ± 0.02
Isoleucine	1.59 ± 0.03	1.53 ± 0.09
Phenylalanine	1.13 ± 0.04	0.99 ± 0.03
Tyrosine	0.96 ± 0.08	0.70 ± 0.02
Histidine	0.93 ± 0.01	0.82 ± 0.02
Lysine	0.90 ± 0.01	1.19 ± 0.02
Methionine	0.90 ± 0.01	0.73 ± 0.03
Cysteine	0.18 ± 0.01	0.13 ± 0.01
Imino acids (Hyp + Pro)	18.47 ± 0.19 ^a^	17.41 ± 0.13 ^b^
Total Amino Acids	100	100

All data expressed as mean ± S.D. (n = 2). Similar superscript letters within each row were not significantly different (*p* > 0.05).

## Data Availability

Not applicable.

## Supplementary Materials

The following are available online, Figure S1: The schematic diagram for collagen separation using ultrafiltration membrane.
