# Supplementary material for: Comparative Study of The Yield and Physicochemical Properties of Collagen from Sea Cucumber (Holothuria scabra), Obtained through Dialysis and the Ultrafiltration Membrane
Source: Molecules. 2021 Apr 28;26(9):2564. doi: 10.3390/molecules26092564 (PMC8124349; doi:10.3390/molecules26092564)

SUPPLEMENTARY DATA

Figure S1. The schematic diagram for collagen separation using ultrafiltration membrane

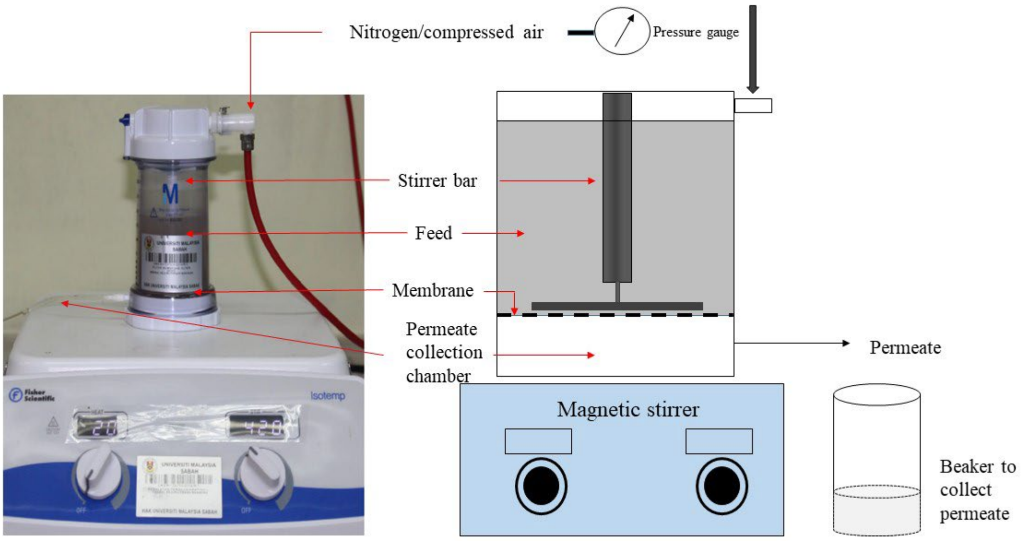

Supplement: Supplementary file 1 [file molecules-26-02564-s001.zip › molecules-1182977-supplementary.pdf]
